# Substrate scope of a dehydrogenase from *Sphingomonas* species A1 and its potential application in the synthesis of rare sugars and sugar derivatives

**DOI:** 10.1111/1751-7915.13272

**Published:** 2018-04-26

**Authors:** Barbara Beer, André Pick, Manuel Döring, Petra Lommes, Volker Sieber

**Affiliations:** ^1^ Chair of Chemistry of Biogenic Resources Technical University of Munich Schulgasse 16 94315 Straubing Germany; ^2^ Catalysis Research Center Technical University of Munich Ernst‐Otto‐Fischer‐Str. 1 85748 Garching Germany; ^3^ Fraunhofer Institute of Interfacial Engineering and Biotechnology (IGB) Bio‐, Electro‐ and Chemo Catalysis (BioCat) Branch Schulgasse 11a Straubing 94315 Germany; ^4^ School of Chemistry and Molecular Biosciences The University of Queensland 68 Cooper Road St. Lucia 4072 Qld Australia

## Abstract

Rare sugars and sugar derivatives that can be obtained from abundant sugars are of great interest to biochemical and pharmaceutical research. Here, we describe the substrate scope of a short‐chain dehydrogenase/reductase from *Sphingomonas* species A1 (SpsADH) in the oxidation of aldonates and polyols. The resulting products are rare uronic acids and rare sugars respectively. We provide insight into the substrate recognition of SpsADH using kinetic analyses, which show that the configuration of the hydroxyl groups adjacent to the oxidized carbon is crucial for substrate recognition. Furthermore, the specificity is demonstrated by the oxidation of d‐sorbitol leading to l‐gulose as sole product instead of a mixture of d‐glucose and l‐gulose. Finally, we applied the enzyme to the synthesis of l‐gulose from d‐sorbitol in an *in vitro* system using a NADH oxidase for cofactor recycling. This study shows the usefulness of exploring the substrate scope of enzymes to find new enzymatic reaction pathways from renewable resources to value‐added compounds.

## Introduction

Sugars and sugar derivatives play an important role in biochemical research, pharmaceutical drug discovery and the food industry as they are involved in a myriad of metabolic processes, including signalling, cellular recognition and processes that are central to human health. However, only a small number of all possible monosaccharides, that is d‐glucose, d‐galactose, d‐mannose, d‐fructose, d‐xylose, d‐ribose and l‐arabinose, according to the International Society of Rare Sugars (Granström *et al*., 2004), are found in nature in sufficient amounts to allow their commercial exploitation. Consequently, the so‐called rare sugars have to be produced by (bio)chemical processes starting from cheap and widely available substrates. Four enzyme classes that can be used for rare sugar production are aldolases, keto–aldol isomerases, epimerases and oxidoreductases (Izumori, 2006; Li *et al*., 2015). Included in the last enzyme class are dehydrogenases/reductases (EC 1.1.‐.‐), which catalyse oxidations and corresponding reductions using different redox‐mediating cofactors. They can be classified into short‐ (SDR), medium‐ (MDR) and long‐chain (LDH) dehydrogenases/reductases according to the number of amino acids. SDRs consist of 250 to 300 amino acids and contain two domains; a cofactor‐binding domain and a substrate‐binding domain. While the cofactor‐binding domain is usually conserved, the substrate‐binding domain varies and determines the substrate specificity (Kavanagh *et al*., 2008). SDR family enzymes use NADP(H) or NAD(H) as a cofactor and substrates include important metabolites such as sugars and their derivatives in carbohydrate metabolism (Koropatkin and Holden, 2005; Zhang *et al*., 2009), steroids in signal transduction (Benach *et al*., 2002; Svegelj *et al*., 2012), and keto‐acyl‐(acyl carrier proteins) and enoyl‐acyl‐(acyl carrier proteins) in fatty‐acid synthesis (Kim *et al*., 2011; Blaise *et al*., 2017). More than 170 000 enzymes in the SDR family are registered in UniProtKB (January 2018). New members of this superfamily are identified every year (there were almost 416 000 new entries in UniProt in 2017), further increasing the vast number of substances known to be metabolized.

Apart from their physiological role, many SDR enzymes are also known for their broad substrate scope (Hirano *et al*., 2005; Pennacchio *et al*., 2010; Stekhanova *et al*., 2010; Ghatak *et al*., 2017; Roth *et al*., 2017). These non‐native substrates can represent interesting starting points for products that are difficult to synthesize chemically. To identify these activities, enzymes can be isolated and exposed to reaction conditions or substrates not seen *in vivo* that will challenge their specificity, possibly forcing them to act on substrates they were not originally designed for. This lack of specificity of enzymes has become increasingly important in white biotechnology for the environmentally friendly synthesis of fine as well as bulk chemicals using enzymes as catalysts (Arora *et al*., 2014). Here, new enzymatic activities are often required for the synthesis of non‐native substances or for the conversion of non‐native intermediates of an artificial metabolic pathway for both *in vivo* and *in vitro* applications. In the latter, with no constraints such as cell viability or transportation across cell boundaries, non‐native substrates can be applied in a straightforward manner. Moreover, side reactions, which can be a disadvantage of using enzymes with lower specificity, can be controlled more easily *in vitro*, as only a limited and specified number of substances, that is, the substrate, the product, and possibly intermediates, are involved.

A NADH/NADPH‐dependent SDR involved in alginate metabolism for the reduction in 4‐deoxy‐l‐erythro‐5‐hexoseulose (DEHU) to 2‐keto‐3‐deoxy‐d‐gluconate (KDG) has been identified in *Pseudomonas* (Preiss and Ashwell, 1962) and *Vibrio splendidus* (Wargacki *et al*., 2012), and recently characterized in detail from *Sphingomonas* species A1 (Takase *et al*., 2010, 2014), *Flavobacterium* species strain UMI‐01 (Inoue *et al*., 2015), and abalone (Mochizuki *et al*., 2015). The last two studies also addressed the question of substrate scope. However, no activity was observed for the reduction in other substrates apart from DEHU. Thus far, no study has examined the reverse reaction (the oxidation reaction) and challenged the specificity with non‐native concentrations of substrates. Here, we provide a comprehensive picture of the substrate scope and specificity of the NAD^+^‐dependent SDR from *Sphingomonas* species A1 (SpsADH, ID: SPH3227, PDB: 4TKM) for oxidation reactions. This enzyme consists of 258 amino acids, has 64% sequence similarity to its NADP^+^‐dependent isoenzyme, and high sequence identity (40%–71%) to 3‐ketoacyl‐CoA‐reductases. Some of the reactions we observed have not been found in nature and may be useful for the synthesis of rare sugars and sugar derivatives.

## Results

### Substrate scope

#### Oxidation of C6 of aldonates

**Figure 1:**
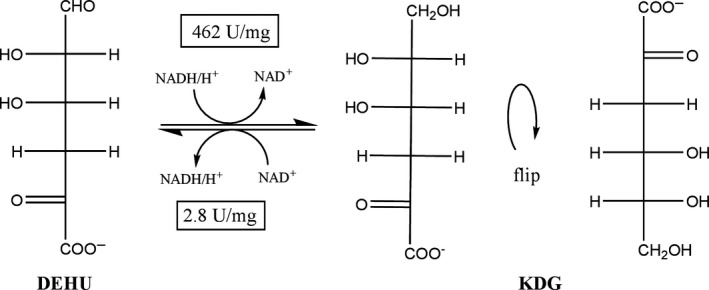
Native reaction of SpsADH. SpsADH is involved in alginate metabolism for the reduction in 4‐deoxy‐l‐erythro‐5‐hexoseulose (DEHU) to 2‐keto‐3‐deoxy‐d‐gluconate (KDG).

SpsADH has been described for the reduction in DEHU to KDG, by reducing the terminal aldehyde (C6 in respect to KDG, Fig. 1). However, we were interested in oxidation reactions rather than reductions. Therefore, we first analysed SpsADH with its native substrate KDG. As expected from thermodynamics, the oxidation of KDG to DEHU was far less preferred compared to reduction, with a *V*_max_ of 2.8 ± 0.2 U mg^−1^ and a *K*_m_ of 13 ± 3 mM. For comparison, Takase *et al*. (2014) reported for the reduction in DEHU a *V*_max_ of 462 ± 33 U mg^−1^ and a *K*_m_ of 4.8 ± 0.6 mM. We then measured the kinetic parameters for the analog d‐gluconate, which has hydroxyl groups at C2 and C3 and obtained similar values as for KDG: *V*_max_ = 2.0 ± 0.1 U mg^−1^; *K*_m_ = 12 ± 2 mM. Encouraged by these positive results, we investigated the substrate specificity of SpsADH. Therefore, we synthesized and performed kinetic analyses with all C6‐ and C5‐d‐aldonates (except d‐idonate, and d‐gulonate, as neither the corresponding aldose nor the aldonates were commercially available) (Table 1). The catalytic efficiency (*k*_cat_/*K*_m_) for the various substrates compared to KDG followed this order: d‐gluconate (79%), d‐mannonate (37%), d‐allonate (10%), d‐altronate (8%), d‐talonate (3%), d‐xylonate (1%), d‐ribonate (0.3%), and d‐arabinonate, d‐lyxonate, and d‐galactonate (0.2%). The formation of the corresponding uronates could be proved by HPLC using the PMP method (Fig. S1). An activity with shorter aldonates, namely d‐erythronate and d‐threonate as well as d‐glycerate was barely measurable; therefore, no kinetic analysis was possible. In addition, the evaluation of the reverse reactions was not possible, as the corresponding uronates are not commercially available or come in too low quantities.

**Table 1:** Kinetic parameters of SpsADH with various substrates. The kinetic parameters of SpsADH with d‐aldonates with a carbon chain length of four to six were determined. Furthermore, polyols with a carbon chain length of three to six were investigated

Structure	Substrate	*K*_m_ (mM)	*V*_max_ (mU mg^−1^)	*k*_cat_ (s^−1^)	*k*_cat_/*K*_m_ (mM^−1^ s^−1^)	*k*_cat_/*K*_m_ (%)
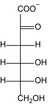	KDG	13 ± 3	2.8 × 10^3^ ± 0.2 × 10^3^	1.4 ± 0.08	10 × 10^−2^ ± 3 × 10^−2^	100
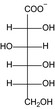	d‐gluconate	12 ± 1	2.0 × 10^3^ ± 0.6 × 10^3^	9.8 × 10^−1^ ± 0.3 × 10^−1^	8.2 × 10^−2^ ± 0.9 × 10^−2^	79
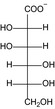	d‐mannonate	14 ± 1	1.1 × 10^3^ ± 0.2 × 10^3^	5.3 × 10^−1^ ± 0.1 × 10^−1^	3.8 × 10^−2^ ± 0.4 × 10^−2^	37
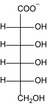	d‐allonate	16 ± 2	3.6 × 10^2^ ± 0.1 × 10^2^	1.8 × 10^−1^ ± 0.05 × 10^−1^	1.1 × 10^−2^ ± 0.2 × 10^−2^	10
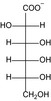	d‐altronate	24 ± 4	3.9 × 10^2^ ± 0.2 × 10^2^	1.9 × 10^−1^ ± 0.08 × 10^−1^	8.0 × 10^−3^ ± 2 × 10^−3^	8
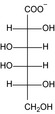	d‐galactonate	50 ± 5	16 ± 1	7.9 × 10^−3^ ± 0.3 × 10^−3^	1.6 × 10^−4^ ± 0.2 × 10^−4^	0.2
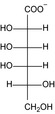	d‐talonate	6 ± 1	42 ± 2	2.1 × 10^−2^ ± 0.1 × 10^−2^	3.4 × 10^−3^ ± 0.9 × 10^−3^	3
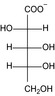	d‐ribonate	16 ± 1	10 ± 0.2	5.2 × 10^−3^ ± 0.1 × 10^−3^	3.3 × 10^−4^ ± 0.3 × 10^−4^	0.3
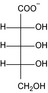	d‐arabinonate	27 ± 3	10 ± 0.3	4.8 × 10^−3^ ± 0.1 × 10^−3^	1.8 × 10^−4^ ± 0.2 × 10^−4^	0.2
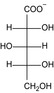	d‐xylonate	24 ± 2	66 ± 4	3.2 × 10^−2^ ± 0.2 × 10^−2^	1.4 × 10^−3^ ± 0.2 × 10^−3^	1
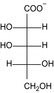	d‐lyxonate	27 ± 2	8.6 ± 0.3	4.2 × 10^−3^ ± 0.1 × 10^−3^	1.6 × 10^−4^ ± 0.2 × 10^−4^	0.2
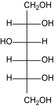	d‐sorbitol	3.5 × 10^2^ ± 0.1 × 10^2^	11 ± 0.2	5.2 × 10^−3^ ± 0.09 × 10^−3^	1.5 × 10^−5^ ± 0.08 × 10^−5^	0.01
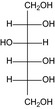	d‐mannitol	1.1 × 10^2^ ± 0.1 × 10^−2^	5.9 ± 0.3	2.9 × 10^−3^ ± 0.1 × 10^−3^	2.6 × 10^−5^ ± 0.4 × 10^−5^	0.03
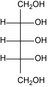	d‐ribitol	16 × 10^2^ ± 4 × 10^−4^	45 ± 6	2.3 × 10^−2^ ± 0.3 × 10^−2^	1.5 × 10^−5^ ± 0.5 × 10^−5^	0.01
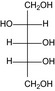	d‐arabitol	2.2 × 10^2^ ± 0.2 × 10^2^	39 ± 1	19 × 10^−3^ ± 0.6 × 10^−3^	8.7 × 10^−5^ ± 1 × 10^−5^	0.08
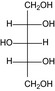	d‐xylitol	14 × 10^2^ ± 2 × 10^2^	16 ± 1	7.8 × 10^−3^ ± 0.6 × 10^−3^	5.5 × 10^−6^ ± 1 × 10^−6^	0.01
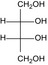	*meso*‐Erythitol	7.0 × 10^2^ ± 1 × 10^2^	39 ± 3	1.9 × 10^−2^ ± 0.1 × 10^−2^	2.7 × 10^−5^ ± 0.6 × 10^−5^	0.03
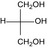	Glycerol	1 × 10^2^ ± 0.1 × 10^2^	11 ± 0.4	5.6 × 10^−3^ ± 0.2 × 10^−3^	2.7 × 10^−5^ ± 0.6 × 10^−5^	0.04

### Oxidation of polyols

**Figure 2:**
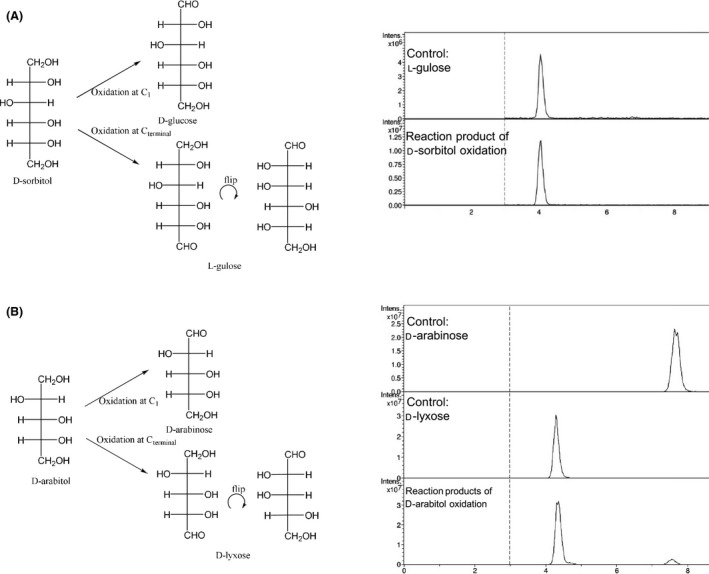
Product identification of d‐sorbitol and d‐arabitol oxidation. In polyols, two primary hydroxyl groups are present, which could be oxidized by SpsADH, which would lead to different aldoses. For d‐sorbitol, only l‐gulose was detected (A), for d‐arabitol (B), both d‐lyxose as well as traces of d‐arabinose were measurable.

The next group of substrates we tested was polyols. Here, the catalytic efficiencies for the tested polyols glycerol, meso‐erythritol, d‐ribitol, d‐arabitol, d‐xylitol, d‐mannitol, and d‐sorbitol were extremely low ranging from 8.7 × 10^−5^ to 1.5 × 10^−5^ for d‐arabitol and d‐sorbitol respectively (Table 1). As two primary hydroxyl groups are present in these molecules, here termed C_1_ and C_terminal_, that can be oxidized to form different aldoses, we analysed the products of the oxidation of d‐sorbitol and d‐arabitol using the PMP method (see Experimental section). The oxidation of d‐sorbitol resulted in l‐gulose as the sole product, which means that only C_terminal_ was oxidized by SpsADH. d‐arabitol oxidation yielded d‐lyxose as well as traces of d‐arabinose (Fig. 2). The resulting aldoses of the other polyols have either d‐ or l‐configuration depending on the carbon being oxidized. These could not be distinguished with this analytical method. The reverse reactions, that is, the reduction in aldoses was not measurable with C5 and C6 sugars, which is unsurprising, as the reduction in the aldehyde requires adoption by the aldoses of an open‐chain configuration. Accordingly, the C4 sugars were better substrates. Here, only the activity at a fixed substrate concentration, 25 mM, was compared as follows: d‐erythrose: 5 mU mg^−1^, d‐threose: 43 mU mg^−1^, d‐glyceraldehyde: 7 mU mg^−1^, and l‐glyceraldehyde: 346 mU mg^−1^.

Other aldehydes lacking hydroxyl groups such as propanal, butanal, hexanal and 4‐oxobutanoic acid were not reduced by SpsADH. However, pyruvaldehyde, carrying a keto‐group, and *iso*‐butanal, having a methyl‐side‐chain at the carbon adjacent to the carbon being oxidized, were accepted with an activity of 25 mU mg^−1^ and 5.5 mU mg^−1^, respectively, at 25 mM substrate concentration each.

### Stability and total turnover

As SpsADH exhibits only very low activities towards non‐native substrates, the stability of the enzyme is very important to ensure complete conversion by extended incubation. Therefore, we analysed the enzyme at 25°C in ammonium bicarbonate buffer pH 7.9 with 1 mM NAD^+^ and found the half‐life to be 20 h. In the case of oxidation of d‐gluconate to l‐guluronate, this gives a total turnover number of 28.300.

### Production of l‐gulose from d‐sorbitol

**Figure 3:**
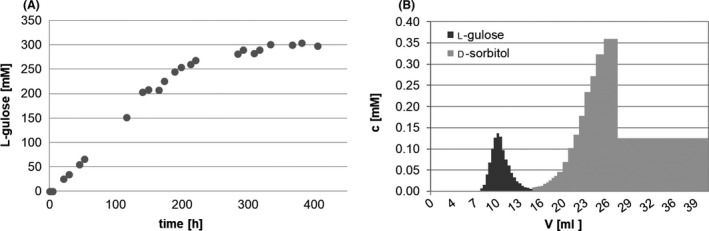
Synthesis of l‐gulose using SpsADH. A. Starting from d‐sorbitol, 300 mM l‐gulose was produced by SpsADH within 234 h. This gives a space‐time yield of 1.3 mM h^−1^. B. Purification of l‐gulose by cation exchange chromatography. Multiple measurements were not conducted as this is only a proof‐of‐concept experiment.

We applied SpsADH for the synthesis of the rare and high‐priced sugar l‐gulose from available and cheap d‐sorbitol in a proof‐of‐concept experiment. From the kinetic analysis, we found an extremely high *K*_m_ of 350 ± 20 mM and only a low *V*_max_ of 11 ± 0.2 mU mg^−1^. Nonetheless, within 230 h, 300 mM l‐gulose were produced, resulting in a space‐time yield (STY) of 1.3 mM h^−1^ (4.0 g L^−1^ d^−1^) (Fig. 3A). Although the theoretical *V*_max_ was not reached here, as this value only reflects the activity without any product present, SpsADH seemed to be stabilized by sorbitol, as it has also been reported for various other proteins (Brennan *et al*., 2003; Khajehzadeh *et al*., 2016; Pazhang *et al*., 2016). This allowed SpsADH to be active for an extended time period of approximately 230 h, resulting in this high yield.

During the purification procedure, glucose was found as an impurity of the d‐sorbitol charge. Due to the similar properties of d‐glucose and l‐gulose, we converted d‐glucose to d‐gluconate using glucose dehydrogenase and catalase. Gluconate was then separated from l‐gulose and d‐sorbitol by anion exchange chromatography with a recovery of 95% of the l‐gulose/d‐sorbitol mixture. Subsequent purification of l‐gulose was possible by cation exchange chromatography with a recovery of 66% pure l‐gulose (Fig. 3B).

## Discussion

‘Where there is oxidation there will always be reduction’ (Robins and Osorio‐Lozada, 2012) is essentially the same as: where there is reduction, there will always be oxidation. The native reaction of SpsADH is the reduction in DEHU to KDG in alginate metabolism. Although this reaction is highly important for third‐generation biomass utilization, where degradation of alginate from brown macro algae, for instance, plays a key role (Wargacki *et al*., 2012; Mochizuki *et al*., 2015), we were interested in the oxidation reaction, and beyond this, in the oxidation of other substrates apart from KDG.

The oxidation of KDG to DEHU is unfavourable compared to the reduction due to thermodynamics, and possibly also due to the physiological role of SpsADH as DEHU was indicated to be toxic to cells and therefore needs to be metabolized to non‐hazardous KDG (Hashimoto *et al*., 2010). Nonetheless, oxidation is possible at pH 8 and furthermore, SpsADH can also oxidize the analog d‐gluconate with only 21% reduced catalytic efficiency. Here, C2 and C3 carry hydroxyl groups, whereas C1, C4, C5 and C6 are the same as in KDG. Interestingly, other C6‐aldonates of the erythro‐group, that is d‐allonate, d‐altronate, and d‐mannonate, which differ only in the configuration of the hydroxyl groups at C2 and C3, show a sharp decrease in the catalytic efficiency. For d‐aldonates from the threo‐group (d‐talonate and d‐galactonate), as well as C5‐d‐aldonates, the catalytic efficiency decreases even further. A general rule to substrate recognition is that the carbon adjacent to the carbon being oxidized needs to carry a substituent (hydroxyl‐, keto‐, or methyl‐group). Furthermore, from the specific oxidation of d‐sorbitol to l‐gulose instead of d‐glucose, we conclude that the adjacent hydroxyl‐group has to be in the d‐conformation. This is also true for the reduction in glyceraldehyde, where the l‐enantiomer is clearly preferred over d‐glyceraldehyde, meaning that in the reverse reaction, glycerol only binds when the hydroxyl group at C2 is positioned in l‐configuration. Furthermore, the position of the hydroxyl group at the next but one carbon also seems to play a role in substrate recognition, as d‐arabitol was mainly oxidized to d‐lyxose with only traces of d‐arabinose. It also shows in the preference for C6‐d‐aldonates of the erythro group over those of the threo‐group. In general, the substrates are better recognized, if they are anionic: the catalytic efficiencies of aldonates always exceed those of the corresponding polyols. For d‐gluconate, it is even about 5500 times higher than for the corresponding polyol d‐sorbitol (8.2 × 10^−2^ and 1.5 × 10^−5^ mM^−1^ s^−1^ respectively). Even though the activity was so low, we were able to produce the rare sugar l‐gulose from d‐sorbitol in an *in vitro* system with a STY of 4.0 g L^−1^ d^−1^. In comparison, another single‐step conversion from d‐sorbitol to l‐gulose using a mannitol dehydrogenase from *Apium graveolens* in a recombinant *Escherichia coli* strain resulted in a STY of 0.9 g L^−1^ d^−1^ (Woodyer *et al*., 2010). Furthermore, a glucose 6‐phosphate isomerase from *Pyrococcus furiosus* was also assessed for the production of l‐gulose from l‐idose; however, incomplete conversion as well as formation of the side‐product l‐sorbose, is downsides of this reaction (Yoon *et al*., 2009).

From other polyols tested here, the rare sugars l‐erythrose (from meso‐erythritol), l‐ribose (from d‐ribitol), d‐lyxose (from d‐arabitol) or l‐xylose (from d‐xylitol) could be obtained.

The aldonates can be converted to uronic acids, which could be further oxidized by uronate dehydrogenases (UDHs) to the corresponding aldaric acids, which are considered top value‐added chemicals to be obtained from biomass (Werpy and Petersen, 2004). They can, for example, be used as building blocks for polymers and hyperbranched polyesters, and also for the metabolite α‐ketoglutarate, for which an *in vitro* enzymatic cascade reaction has been established (Beer *et al*., 2017). Here, glucuronate was used as a substrate, but glucose could also be converted using glucose dehydrogenase, SpsADH and UDH. However, so far, UDHs are only known to participate in the oxidation of d‐glucuronate, d‐galacturonate and d‐mannuronate (Pick *et al*., 2015). Therefore, engineering of UDH, so that it is active with l‐guluronate, is necessary here.

In summary, we showed that SpsADH exhibits a broad substrate scope, when challenged with non‐native substrate concentrations. Research groups that previously investigated the substrate specificity of other DEHU reductases could not find any activity at a 1 mM substrate concentration (Inoue *et al*., 2015; Mochizuki *et al*., 2015). We believe that these enzymes could also show activity, if higher concentrations of substrates were used. Even with the very low (mU mg^−1^ range) activities found here, the long half‐life of SpsADH allows conversion of significant amounts of substrate. In addition, these activities can be the starting point for enzyme engineering to enhance specific activities. The availability of the crystal structure (4TKM, Takase *et al*., 2014) will facilitate this, as substrates of interest can be docked *in silico* into the binding pocket to find possible amino acid positions to be exchanged.

## Experimental procedures

### Reagents

Restriction enzymes, alkaline phosphatase, T4 ligase and Taq polymerase were obtained from New England Biolabs. Oligonucleotides were from biomers.net GmbH. All chemicals were of analytical grade or higher quality and purchased from Carbosynth, Sigma‐Aldrich, Alfa Aesar, Serva, or Carl Roth. For protein purification, equipment (including columns) from GE Healthcare was used.

### Preparation of aldonic acids and dehydrated aldonic acids

Aldonic acids were prepared from their corresponding sugars by oxidation using a gold catalyst (J213 XI/D 0.5%, 0.5% Au on Al_2_O_3_ powder, kindly provided by Evonik Industries, Germany). The procedure has been previously described for l‐arabinose, d‐xylose, d‐galactose and d‐glucose (Sperl *et al*., 2016), as well as for d‐ribose, d‐lyxose, d‐mannose, l‐rhamnose and *N*‐acetyl‐glucosamine (Mirescu and Prüße, 2007), and could be adapted for the oxidation of d‐allose, d‐altrose, d‐talose, d‐arabinose, d‐erythrose, d‐threose, 2‐deoxy‐d‐glucose and 3‐deoxy‐d‐glucose. The results can be found in the supporting information. The procedure was as follows: reactions were carried out in a temperature controlled glass reactor (total volume 50 ml, initial reaction volume 30 ml) equipped with a reflux condenser, a pH electrode and a burette for base dosage, a 21 G needle as the oxygen inlet, and a magnetic stirrer. Prior to the start of the reaction, the sugar solution was stirred at the desired pH value inside the reactor. This solution was heated to 50°C and oxygen was supplied at a flow rate of 40 ml min^−1^. The reactions were then initiated by adding an appropriate amount of gold catalyst (see Supporting Information). During the reaction, the oxygen supply was maintained at the same flow rate and the pH value was kept constant with an automatic titrator (TitroLine 7000, SI Analytics, Germany) by adding aqueous NaOH. During the course of the reaction, the amount of added NaOH was monitored. Conversion and selectivity were checked with a HPLC system as described below.

For kinetic measurements, the aldonic acids were purified (preparative scale 200–1080 mg). For this, the resulting solution was loaded to a Dowex (1 × 8, 200–400 mesh) anion exchange column, which was equilibrated with ammonium bicarbonate (AbC). The column was washed with three column volumes (CV) of H_2_O and the sugar acids were eluted with 100 mM AbC (pH 10.4). The pooled fractions containing the sugar acids were lyophilized and dissolved in H_2_O to give a concentration of 10 mg ml^−1^. This solution was loaded to a Dowex (50Wx8‐400, 200–400 mesh) cation exchange column in the Na^+^‐form. Elution with H_2_O and lyophilization gave the sugar acids as the sodium salt. Karl Fischer titrations were performed to analyse the water content and to calculate standard concentrations accordingly.

The keto‐deoxy sugar KDG was obtained from d‐gluconate using the enzyme dihydroxy‐acid dehydratase from *Sulfolobus solfataricus*. This detailed procedure is described by Sperl *et al*. (2016).

### Construction of plasmid for overexpression

The DNA sequence encoding alcohol dehydrogenase from *Sphingomonas* species A1 (SpsADH, GenBank™ accession number BAP40335.1) was synthesized as a GeneArt^®^Strings DNA fragment with optimized codon usage for expression in *E. coli* and restrictions sites for NdeI and XhoI (Life Technologies). The fragment was first cloned into pJET1.2 by blunt end ligation using a kit from Thermo Fisher Scientific. The gene was then cloned into pET‐28a(+) (Novagen) with an N‐terminal His_6_‐tag yielding the plasmid pET‐28a‐NHis‐*spsadh*. Multiplication of the plasmids was performed in *E. coli* DH5α (Stratagene) in LB medium containing 30 μg ml^−1^ kanamycin.

### Expression and purification

For small amounts, expression of *spsadh* was performed in *E. coli* BL21(DE3) (Novagen) containing the plasmid of interest in 1 L autoinduction medium that included 100 μg ml^−1^ kanamycin (Studier, 2005). The preculture was incubated in 20 ml LB medium with 30 μg ml^−1^ kanamycin at 37°C overnight on a rotary shaker (180 rpm). Expression cultures were inoculated with the overnight culture at an OD_600_ of 0.1. Incubation was performed in a shaking flask for 3 h at 37°C followed by incubation for 21 h at 16°C on a rotary shaker (120 rpm). Cells were harvested by centrifugation and stored at −20°C. For large amounts, expression of *spsadh* was performed using a 2 L Biostat Cplus bioreactor (Sartorius Stedim, Germany) in a batch cultivation process. The fermentation parameters were as follows: 2 L autoinduction medium containing 100 μg ml^−1^ kanamycin was inoculated with 10% of a preculture grown overnight. The temperature was set at 37°C until an OD_600_ of 9 was reached, then it was decreased to 16°C for 24 h. The pH was held constant at 7 and the pO_2_ at 30% with variable stirring speed and aeration. Cells were harvested by centrifugation and stored at −20°C.

For purification of SpsADH, cells were resuspended in 50 mM sodium phosphate buffer (pH 8.0, 20 mM imidazole, 500 mM NaCl, and 10% glycerol). Crude extracts were prepared on ice by ultrasonication (Hielscher Ultrasonics, sonotrode LS24d10): three cycles of 15‐min pulsing (0.6 ms, 0.4 ms pause) at 80% amplitude. The insoluble fraction of the lysate was removed by centrifugation (20 000 rpm for 40 min at 4°C), and the supernatant was then applied to an IMAC affinity resin column (5 ml HisTrap™ FF) equilibrated with the resuspension buffer using the ÄKTA Purifier system. The column was washed with five column volumes of resuspension buffer and the His‐tagged protein was eluted by a gradient of 10 column volumes of 0% to 100% elution buffer (50 mM sodium phosphate buffer; pH 8.0, 500 mM imidazole, 500 mM NaCl, and 10% glycerol). Aliquots of the eluted fractions were subjected to 12% SDS‐PAGE as described by Laemmli (1970). The molecular weight was calculated using the ProtParam tool (Gasteiger 2005): this was 29 500 Da, including the additional amino acids of the N‐terminal His_6_‐tag. The fractions containing the eluted protein were pooled and the protein was desalted using a HiPrep™ 26/10 Desalting column, which was preliminarily equilibrated with 50 mM AbC, pH 7.9. The protein concentration was determined by UV spectroscopy (NanoPhotometer, Implen) at 280 nm using the extinction coefficient (ε_280_) calculated using the ProtParam tool (assuming all cysteines are reduced): 18 450 M^−1 ^cm^−1^. Aliquots of the protein solution were shock‐frozen in liquid nitrogen and stored at −80°C.

### Enzyme assays

The activity of SpsADH was determined photometrically by monitoring the increase/decrease in NADH at 340 nm using the Infinite 200 PRO photometer (Tecan Group Ltd.). Reactions were performed at 25°C in triplicate using 96‐well microtiter plates. For determination of the kinetic parameters, the reaction mixtures contained 50 mM AbC, pH 7.9, 0.3 mM NADH (or 1 mM NAD^+^), various concentrations of substrate, and an appropriate amount of purified enzyme. The initial reaction velocity was measured for 1 to 2 min and the calculation of Michaelis–Menten kinetics for determination of *K*_m_ and *V*_max_ was carried out using SigmaPlot 11.0 (Systat Software). One unit of enzyme activity was defined as the amount of protein that reduced (oxidized) 1 μmol of NAD(H) min^−1^ at 25°C.

For product identification, enzyme assays were performed overnight with the addition of the cofactor recycling enzyme NADH oxidase (NOX) from *Lactobacillus pentosus*. Expression, purification and activation with FAD were performed as described previously (Nowak *et al*., 2015). Reaction mixtures contained 50 mM AbC, pH 7.9, 25 mM substrate, 5 mM NAD^+^ and purified SpsADH. Fresh NOX was added two more times during the incubation time due to stability issues (Beer *et al*., 2017).

### HPLC analysis

The synthesized aldonic acids were analysed by HPLC, using an UltiMate 3000 HPLC system (Dionex, Idstein, Germany), equipped with autosampler (WPS 3000TRS), a column compartment (TCC3000RS) and a diode array detector (DAD 3000RS). The Metrosep A Supp10–250/40 column (250 mm, particle size 4.6 mm; Metrohm, Filderstadt, Germany) at 65°C was used for separation by isocratic elution with 12 mM AbC, pH 10.0 as the mobile phase at 0.2 ml min^−1^. Samples were diluted in water, filtered (10 kDa MWCO, modified PES; VWR, Darmstadt, Germany), and 10 μl were applied to the column. Data were analysed using Dionex Chromeleon software.

Uronic acids and aldoses were analysed by HPLC/MS as 1‐phenyl‐3‐methyl‐5‐pyrazolone (PMP) derivatives using the method of Rühmann *et al*. (2014).

Samples were diluted in water and derivatized with 0.1 M PMP and 0.4% ammonium hydroxide in methanol. After 100 min at 70°C, the reaction was stopped with 16.7 mM acetic acid and filtered (Restek, 0.22 μm, PVDF). When elution of the uronic acid was expected before 3 min, the sample was extracted with chloroform (three times) to reduce excess PMP and then filtered.

The HPLC system (UltiMate 3000RS, Dionex) was composed of a degasser (SRD 3400), a pump module (HPG 3400RS), an autosampler (WPS 3000TRS), a column compartment (TCC3000RS), a diode array detector (DAD 3000RS) and an ESI ion trap unit (HCT, Bruker). Data were collected and analysed using Bruker HyStar software. The column (Gravity C18, 100 mm length, 2 mm i.d.; 1.8 μm particle size; Macherey‐Nagel) was operated at 50°C with a mobile phase A (5 mM ammonium acetate buffer, pH 5.6, with 15% acetonitrile) and a chromatographic flow rate of 0.6 ml min^−1^. The gradient (mobile phase B containing pure acetonitrile) was as follows: start of mobile phase B at 1%, with an increase to 5% over 5 min, held for 2 min, then an increase to 18% over 1 min. The gradient was further increased to 40% over 0.3 min, held for 2 min, and returned within 0.2 min to starting conditions for 1.5 min. If samples were not extracted, the first 3 min of chromatographic flow were refused by a switch valve behind the UV detector (245 nm). Before entering ESI‐MS, the flow was split 1:20 (Accurate post‐column splitter, Dionex). The temperature of the autosampler was set to 20°C and an injection volume of 10 μl was used.

ESI ion trap parameters: The ion trap operated in the ultra scan mode (26 000 m/z/s) from 50 to 1000 m/z. The ICC target was set to 200 000 with a maximum accumulation time of 50 ms and four averages. The ion source parameters were set as follows: capillary voltage 4 kV, dry temperature 325°C, nebulizer pressure 40 psi, and dry gas flow 6 L min^−1^. Auto MS mode with a smart target mass of 600 m/z and an MS/MS fragmentation amplitude of 0.5 V was used. Analysis was performed using the extracted ion chromatograms of the m/z value corresponding to the protonated molecules.

### Production of l‐gulose from d‐sorbitol

Production of l‐gulose was carried out in a volume of 50 ml in a two‐necked round‐bottom glass flask. One neck was used for drawing samples, and the second for supplementing gaseous, humidified oxygen through a cannula at 20 ml min^−1^ once a day for 1 to 2 min. The reaction mixture contained 50 mM AbC, 1 mM NAD^+^, 1.5 M d‐sorbitol and 0.04 U ml^−1^ of SpsADH and NOX respectively. The kinetic parameters of NOX were determined in the presence of 1.5 M d‐sorbitol in 50 mM AbC, and varying amounts of NADH.

The reaction took place at 25°C (water bath) with stirring. 0.04 U ml NOX was added multiple times over the entire duration of the experiment. Furthermore, to maintain the activity of SpsADH at *V*_max_, 3.0 and 4.5 g d‐sorbitol were added after 117 and 200 h respectively.

Samples for HPLC analysis were ultrafiltrated with spin filters (10 kDa MWCO, modified PES; VWR) to remove enzymes and stop the reaction. The experiment was stopped after there was no further rise in l‐gulose concentrations.

Purification procedure: Due to an impurity of the used d‐sorbitol charge, glucose had to be first removed. The reaction mixture was consequently incubated with glucose oxidase and catalase to convert glucose to gluconate, which was then removed by anion exchange chromatography using a Dowex 1 × 8, 200–400 mesh column. Pure l‐gulose was then obtained by cation exchange chromatography (Dowex 50Wx8‐400, 200–400 mesh column in the Na^+^‐form).

Elution monitoring and quantification was performed using a HPLC system (Dionex, Sunnyvale, CA, USA) equipped with a Rezex ROA‐H^+^ column (Phenomenex, Torrance, CA, USA), and a refractive index detector (RI‐101, Shodex, Tokyo, Japan). The mobile phase (sulphuric acid, 2.5 mM) was set to a flow rate of 0.5 ml min^−1^ at an oven temperature of 70°C. Quantitative calculations were referred to an external standard.

## Conflict of interest

None declared.

## Supporting information


**Table S1.** Synthesis of aldonic acids.
**Fig. S1.** Product identification of aldonate oxidation.
