# Supplementary material for: Substrate scope of a dehydrogenase from Sphingomonas species A1 and its potential application in the synthesis of rare sugars and sugar derivatives
Source: Microb Biotechnol. 2018 Apr 26;11(4):747–58. doi: 10.1111/1751-7915.13272 (PMC6011931; doi:10.1111/1751-7915.13272)
Supplement: Supplementary file 1 — Table S1. Synthesis of aldonic acids. Fig. S1. Product identification of aldonate oxidation. [file MBT2-11-747-s001.docx]

**Substrate scope of a dehydrogenase from *Sphingomonas* species A1 and its potential application in the synthesis of rare sugars and sugar derivatives**

Barbara Beer^[[1]](#footnote-1)^, André Pick^1^, Manuel Döring^1^, Petra Lommes^1^, Volker Sieber^1,^ ^[[2]](#footnote-2), 3, 4^

This work was conducted at the Chair of Chemistry of Biogenic Resources, Technical University of Munich, Schulgasse 16, 94315 Straubing, Germany

Corresponding author: Prof. Dr. Volker Sieber, Technical University of Munich, Schulgasse 16, 94315 Straubing, Germany; phone: +49 9421-187-300, fax: +49 9421-187-310, e-mail: sieber@tum.de, ORCID 0000-0001-5458-9330

*Synthesis of aldonic acids*

The following aldonic acids were synthesized by oxidation of the corresponding sugars using a gold catalyst: D-erythronate, D-threonate, D-arabonate, D-lyxonate, D-ribonate, D-xylonate, D-allonate, D-altronate, D-mannonate, D-galactonate, D-talonate, 2-deoxy-D-gluconate, and 3-deoxy-D-gluconate. The optimal ratios of sugar to catalyst (Table S 1) were found by stepwise addition of the catalyst to the reaction mixture with online monitoring of the reaction using a titrator.

Table S 1: Synthesis of aldonic acids

| Sugar | Au catalyst (mg)/ sugar (mmol) | conversion (%) | water content of product (%) |
| --- | --- | --- | --- |
| D-erythrose | 180 | 92.5 | 11.38 |
| D-threose | 60 | 99.8 | 17.54 |
| D-arabinose | 10 | 99.7 | 2.22 |
| D-lyxose | 27 | 98.3 | 12.16 |
| D-ribose | 30 | 98.8 | 11.93 |
| D-xylose | 20 | 100 | 9.41 |
| D-allose | 60 | 99.3 | 15.37 |
| D-altrose | 40 | 97.1 | 12.60 |
| D-mannose | 30 | 92.0 | 1.94 |
| D-galactose | 8 | 100 | 11.94 |
| D-talose | 60.5 | 93.5 | 12.55 |
| 2-deoxy-D-glucose* | 54.2 | 96.7 |  |
| 3-deoxy-D-glucose* | 75 | 95 |  |

* not optimized

Verification of uronate formation by HPLC using the PMP method


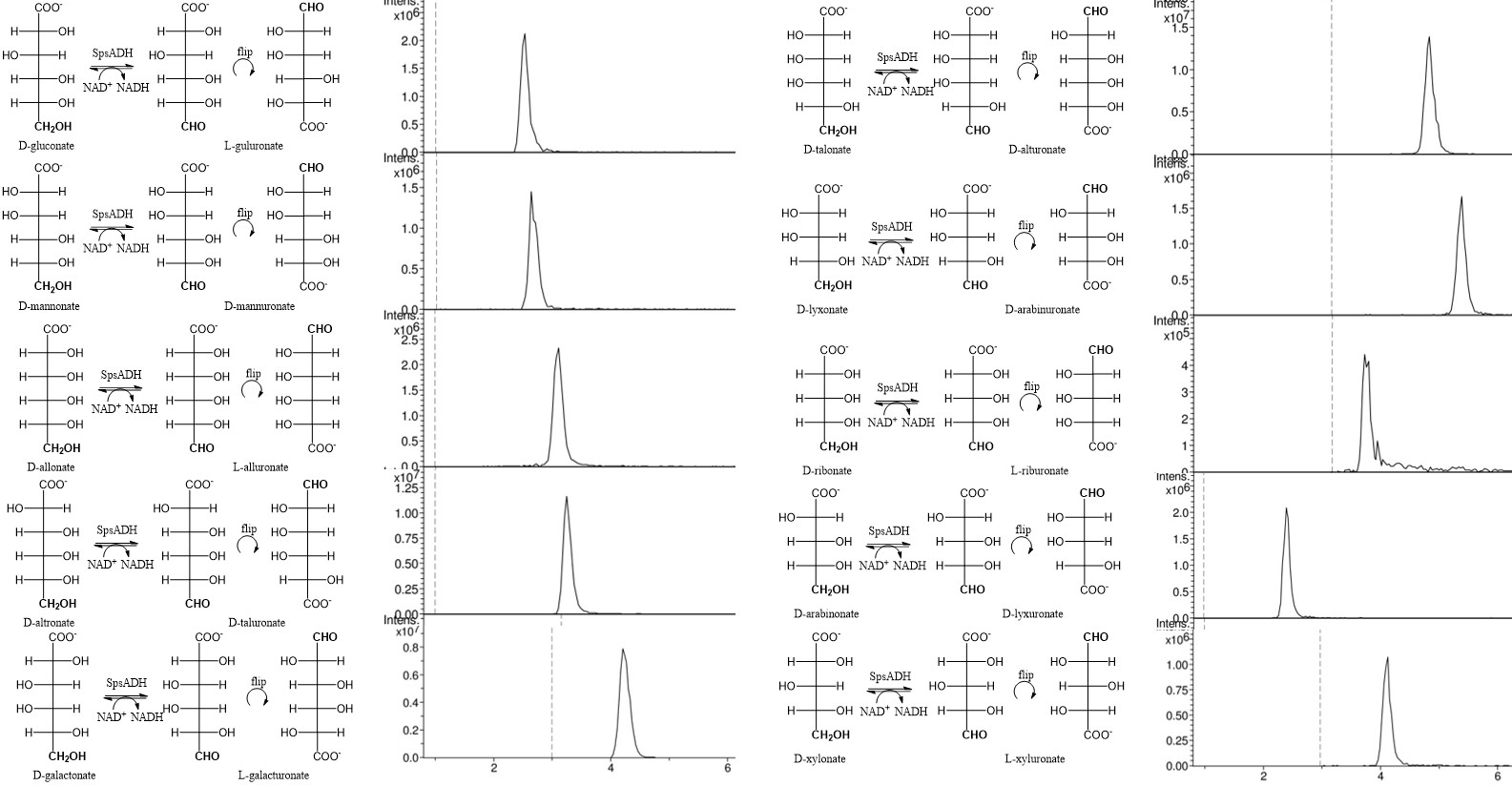


Figure S 1: Product identification of aldonate oxidation

Aldonates are converted to uronic acids by SpsADH. These were derivatized with PMP (see experimental section of main paper) and analyzed by HPLC. The dotted line indicates the valve switch for MS measurement. A valve switch before 3 min was only performed with extracted samples, where access PMP was removed.

1. Chair of Chemistry of Biogenic Resources, Technical University of Munich, Schulgasse 16, 94315 Straubing, Germany [↑](#footnote-ref-1)
2. Chair of Chemistry of Biogenic Resources, Technical University of Munich, Schulgasse 16, 94315 Straubing, Germany

   3 Fraunhofer Institute of Interfacial Engineering and Biotechnology (IGB), Bio-, Electro- and Chemo Catalysis (BioCat) Branch, Schulgasse 11a, Straubing 94315, Germany

   ^4^ The University of Queensland, School of Chemistry and Molecular Biosciences, 68 Cooper Road, St. Lucia, 4072, Australia. [↑](#footnote-ref-2)
